# Mechanistic Insights into Proglumide’s Role in Immune Cell Efficacy and Response to Immune Checkpoint Inhibitor Therapy in Hepatocellular Carcinoma

**DOI:** 10.3390/cancers17182998

**Published:** 2025-09-14

**Authors:** Priyanka S. Doneparthi, Hong Cao, Wenqiang Chen, Wenyu Dou, Hong-Bin Fang, Jill. P. Smith

**Affiliations:** 1 Department of Oncology, Georgetown University, Washington, DC 20007, USA; 2 Department of Medicine, Georgetown University, Washington, DC 20007, USA; 3 Department of Biostatistics, Georgetown University, Washington, DC 20007, USA

**Keywords:** hepatocellular carcinoma, tumor microenvironment, T-cells, microbiome, immune checkpoint inhibitors

## Abstract

Background: New strategies are needed to improve the response to immune checkpoint inhibitors for the treatment of hepatocellular carcinoma. Methods: Mice bearing HCC tumors were treated with PBS (control), a PD-1 antibody (PD-1Ab), proglumide, or the combination of proglumide and the PD-1Ab. The tumor microenvironment (TME) was evaluated histologically for fibrosis and by immunohistochemistry for immune cells. To investigate the mechanisms involved in T-cell efficiency, mouse spleen cells were isolated and examined for T-cell exhaustion markers and cytokine release. The mouse microbiome was analyzed using whole-genome sequencing before therapy and at the end of the study. Results: The combination of proglumide with a PD-1Ab decreased tumoral fibrosis better than monotherapy, and altered the immune cell signature in the TME by decreasing M2-polarized macrophages and increasing the influx of CD8+ T-cells. Proglumide monotherapy or in combination with the PD-1Ab decreased T-cell exhaustion markers and improved cytokine release. The combination therapy resulted in changes to the microbiome, including increased beneficial bacteria and genera known to enhance the efficacy of ICIs. Conclusions: Co-administration of proglumide with ICIs resulted in remodeling of the TME, changing a “cold” tumor to a “hot” immune-responsive tumor, activating T-cells, and altering the host microbiome to a population of bacteria that are beneficial.

## Simple Summary

Hepatocellular carcinoma (HCC) is the fastest-growing cause of cancer-related death worldwide. Treatment with immune checkpoint inhibitors (ICIs) has shown promise in HCC; however, the response rate remains less than 50%. One reason for the low efficacy is fibrosis in most livers that harbor HCC and in the tumor which prevents the penetration of anti-cancer agents and influx of CD8+ T-cells. In this study, we evaluated the ability of proglumide, an oral cholecystokinin-B receptor antagonist, to improve the efficacy of ICIs using a murine model of HCC.

## 1. Introduction

Currently, hepatocellular carcinoma (HCC) is the fastest growing cause of cancer-related deaths [1]. Infection with chronic hepatitis B or C virus is currently the dominant risk factor worldwide, accounting for more than 1.3 million deaths per year from HCC [2]. However, with the increasing prevalence of obesity, metabolic dysfunction-associated steatohepatitis (MASH) has become a new risk factor for cirrhosis and HCC [3,4]. Once HCC develops, surgical resection or orthotopic transplantation offer the only hope for a cure, but not all patients are candidates for surgery. In advanced HCC, tyrosine kinase inhibitors such as sorafenib, lenvatinib, regorafenib, and cabozantinib may extend survival for a few months but are not curative [5]. Treatment with immune checkpoint inhibitors (ICI) has shown promise in HCC, but the response is only 15–20% with monotherapy and 25–40% with combination therapies [6]. Nivolumab and pembrolizumab, as well as the combination of nivolumab plus ipilimumab, were granted accelerated approval by the FDA for previously treated patients with sorafenib. The combination of atezolizumab and bevacizumab was shown to improve overall survival and progression-free survival compared with sorafenib in a front-line phase III trial [7]. However, gastrointestinal bleeding is an unacceptable side effect of bevacizumab, and ICIs can induce immune-mediated toxicity. New strategies for target-specific HCC therapy with limited toxicity are needed.

The PD-1/PD-L1 pathway plays a pivotal role in immune escape in cancers and in hepatocellular carcinoma [8] and studies have shown a survival advantage in patients with HCC tumors expressing higher levels of PD-L1 [9]. Cirrhosis impairs the effectiveness of ICIs by causing cirrhosis-associated immune dysfunction syndrome (CAIDS) [10]. Patients with CAIDS have elevated levels of pro-inflammatory cytokines, however their immune cells—neutrophils, monocytes, T-cells, and NK cells—exhibit compromised functionality, and the T-cells are ‘exhausted T-cells’ [11]. T-cell exhaustion describes a hyporesponsive state, characterized by decreased expression of effector cytokines and increased expression of inhibitory immune checkpoint receptors leading to a decreased response to ICIs [12]. Strategies to reinvigorate T-cell function has become an active area of research [13].

The adverse microenvironment of the cirrhotic liver with dense fibrosis creates a physical barrier to the influx of T-cells and the stroma cells, or fibroblasts, promote HCC progression by reducing tumor immuno-surveillance, stimulating angiogenesis, release of cytokines and promoting epithelial to mesenchymal transition [14]. Although ICIs unleash checkpoint blockade of tumors, their task is undermined by an overabundance of immunosuppressive myeloid cells and the lack of tumor-infiltrating CD8 T-cells leading to ICI resistance [15]. HCC characteristically utilizes various evasion methods to resist anti-tumor immune responses sustained by tumor metabolism [16]. The liver has natural immune tolerance due to its immunosuppressive polarity which weakens the T-cell-mediated antigenic response [17,18]. Due to the innate immune tolerance or escape mechanism of the liver, this environment promotes the growth of cancer cells and shields them from recognition by the immune system [19].

The cholecystokinin-B receptor (CCK-BR) is not found in normal liver tissue but is expressed in HCC, suggesting that this receptor may be a target for the treatment of HCC [20]. CCK-BRs are also expressed in mouse and human stellate cells and activated myofibroblasts [21,22], where they are in part responsible for collagen deposition and fibrosis. Proglumide is a nonselective cholecystokinin receptor antagonist that induces plasticity in stellate cells and decreases collagen production [23]. Mice fed liver injury diets with 3,5-diethoxy-carbonyl 1,4-dihydrocollidine (0.1%, DDC) [24] or a choline-deficient ethionine (75%, CDE) diet [25] developed liver fibrosis which was prevented by concomitant treatment with proglumide. Our research laboratory previously discovered a synergistic effect between proglumide and PD-1Ab therapy in immunocompetent mice bearing syngeneic murine HCC tumors [26]. In this report, proglumide decreased tumoral fibrosis, which allowed for an influx of CD8+ T-cells and prolonged survival in mice treated with proglumide and PD-1 antibody [26].

Microbiome remodeling plays a vital role in mediating tumor response to immunotherapies targeting PD-1Ab [27]. A previous study found that proglumide therapy modified the gut microbiome of mice fed a high-fat diet, changing the microbial signature to resemble that of a healthy control mouse more closely [28]. In the previous study, two bacterial genera, *Akkermansia* and *Alistipes*, were significantly enriched in mice treated with proglumide. These same bacterial genera have been identified in clinical subjects as being associated with enhanced responsiveness to PD-1Ab therapy [27]. The administration of *Akkermansia* or *Alistipes* has been shown to reduce tumor growth more than fecal microbiota transplantation [29,30].

In the current investigation, we studied the mechanisms of CCK-BR antagonism with proglumide alone or in combination with immune checkpoint PD-1Ab therapy, on HCC tumors in immunocompetent mice. By investigating these potential mechanisms, we discovered new insights on how blocking the CCK-BR signaling pathway aids in remodeling the tumor microenvironment, the activity of T-cells, and the modulation of the gut microbiome to enhance the efficacy of immune checkpoint therapy.

## 2. Materials and Methods

### 2.1. Liver Cancer Cells

Murine RIL-175 hepatocellular carcinoma cells [31] were characterized and provided by Dr. Tim Greten from the National Cancer Institute. The cells were grown in a humidified incubator with 5% CO_2_ in RPMI-1640 medium (Gibco, Life Technologies Corporation, Carlsbad, CA, USA, Cat# 11875093) supplemented with 10% fetal bovine serum (CYTIVA, HyClone, Logan, UT, USA, Cat# SH30910.03) and 1% penicillin/streptomycin (Gibco, Carlsbad, CA, Cat# 15140-163. RIL-175 cells were genetically authenticated and tested at the Animal Health Diagnostic Laboratory, NCI Frederick, Frederick, MD, USA, using the Molecular Testing of Biological Materials Mouse (MTBM-M; #861-1) Test, and all tests were negative for pathogens.

### 2.2. Preliminary Dosing and Cell Number Pilot Experiments

In order to determine the optimal number of RIL-175 cells to inoculate into the immune competent mice and evaluate the growth rate, several pilot studies were conducted. Also 3 different doses of the PD-1Ab were tested to determine the optimal dose to combine with proglumide. In the first pilot study mice (N = 20) received an inoculum of 700,000 RIL-175 cells onto the right and left flanks subcutaneously. After only 1 week all the mice had tumors the animals and were divided into 4 groups of equal tumor size (N = 5 mice each and with a total of 10 tumors per group). The mice were then started on treatment with PBS (controls), high dose PD-1 antibody (Ab) 150 μg × 3 (4 days apart), proglumide (0.1 mg/mL in the drinking water), or a combination of PD-1Ab and proglumide. The tumors were measured with calipers at baseline and each week. In this pilot study, the tumors in the PBS-treated control mice grew very rapidly and after 2 weeks and we were required to euthanize the mice (Supplementary Figure S1A) according to the approved IACUC protocol. In the next series of experiments, we tested a tumor inoculums of 200,000 and 100,000 RIL-175 mouse HCC cells as above subcutaneously into immunocompetent mice. In this experiment we tested a moderate PD-1Ab dose (100 µg) and a lower dose (50 µg) given × 3 (4 days apart as above). Tumor take was slower that in the first cohort of mice injected with 700,000 cells and mice had palpable tumors after 2 weeks. The tumor growth was slightly faster in the mice that had been injected with 200,000 cells compared to those receiving the 100,000 cell inoculum (Supplementary Figure S1B). The tumor response with the moderate PD-1Ab dose (100 µg) was compared to the lower dose (50 µg), and showed that the mice treated with the 50 µg dose had smaller tumors. Based upon these pilot studies, it was decided to inject the mice with 100,000 RIL-175 cells so the tumors would not grow so rapidly and we would be able to assess the effects of proglumide on T-cells, tumors, and the microbiome. We also selected the lower PD-1Ab dose because it appeared to decrease the tumor volume as measured by calipers better than the 100 µg dose.

### 2.3. Study Design and Treatments

**Figure 1:**
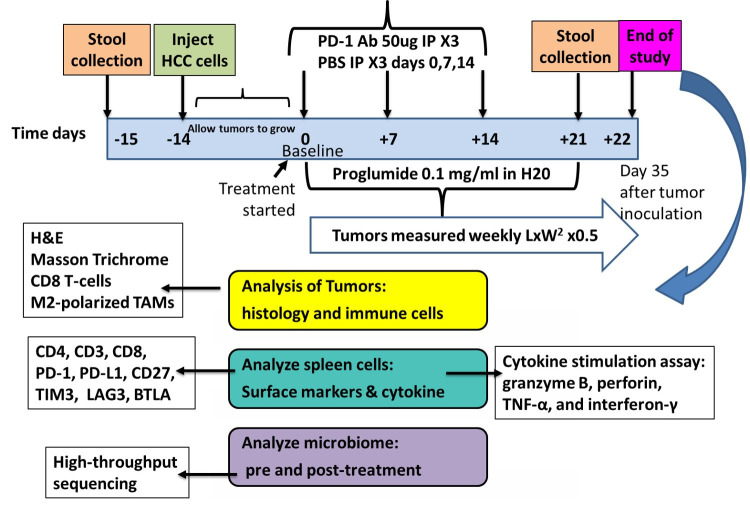
Experimental study design and treatments. C57BL/6 mice were inoculated subcutaneously with 100,000 HCC RIL-175 cancer cells on the right and left flank. When the mice had palpable tumors, they were randomized to one of 4 treatment groups with equal tumor size. Mice received PBS (control), proglumide (0.1 mg/mL in drinking water), PD-1Ab (50 μg, intraperitoneally), or combined therapy with proglumide and PD-1Ab. PD-1Ab and PBS were given on days 0, 7, and 14. PD-1Ab and PBS treatments were given in a volume of 100 μL by the intraperitoneal route. Stool samples were collected before tumor inoculation and the treatment for baseline and at the end of the study for microbiome analysis.

All mouse studies were performed ethically and were approved by the Institutional Animal Care and Use Committee (IACUC) at Georgetown University under the approved protocol (#2023-0031). In the current investigation, twenty male C57BL/6 mice (Charles River Laboratories, Frederick, MD, USA) were injected subcutaneously with RIL-175 HCC cells (100,000) into both the right and left flanks. After two weeks, when the mice had palpable tumors, they were divided into four groups with equal tumor size and treated with the following: Phosphate Buffered Saline (PBS 1x; control; Gibco, Cat#20012-027), proglumide (COSMA S.p.A.; Milan, Italy) at a concentration of 0.1 mg/mL in drinking water, PD-1Ab (Bio X cell, West Lebanon, NH, USA, Cat# BE0146) (50 μg, i.p., on days 0, 7, and 14), and combination therapy with a PD-1Ab and proglumide. Control mice received PBS (100 μL) on the same day as the PD-1Ab was administered. On day 35 after tumor cell inoculation, the mice were euthanized, the spleens collected, and the tumors were excised for histology. A diagram of the study design is shown in Figure 1.

### 2.4. Evaluation of Treatments on Tumor Growth

Growth of the tumors over time was examined weekly using calipers and the formula length × (width)^2^ × 0.5, and body weight was also measured weekly until the day of euthanasia. Tumor volumes reached the maximum allowed size according to the approved IACUC protocol on day 35; hence, the mice were euthanized at this time point by CO_2_ asphyxiation and cervical dislocation.

### 2.5. Spleen T-Cell Isolation

At necropsy, the spleen was removed from each animal and mechanically disrupted as previously described [32] using a 1 mL syringe. In brief, the spleen suspension was filtered through a 70 μm cell strainer, rinsed, and centrifuged at 1500 rpm for 10 min at 4 °C to pellet the cells. The cell pellet was resuspended in RBC lysis buffer, vortexed and incubated for 10 min at room temperature. The reaction was stopped by adding 20 mL of 1X PBS (without EDTA, Ca, and Mg). After more washes in PBS, lymphocytes were pelleted and resuspended in 5 mL of complete medium DMEM (Gibco, Life Technologies Corporation, Carlsbad, CA, USA, Cat#11965-092) with 10% fetal bovine serum and 1% penicillin/streptomycin for cell resuspension for counting.

### 2.6. Analysis of the Tumor Microenvironment by Histology and Immunohistochemistry

The tumors were excised and weighed, and were fixed in 4% paraffin formaldehyde (diluted from Formaldehyde, Avantor Macron Fine Chemicals, Philadelphia, PA, USA, Cat#5016-08) and embedded in paraffin. Tissue sections (5 μm) were mounted on slides for staining with Masson’s trichrome to analyze intratumoral fibrosis. Images from each slide were captured using an Olympus BX43F microscope (Tokyo, Japan) with a Olympus XC50 camera (Japan) Tumors were also stained for CD8+ T-cells and tumor-associated macrophages (TAMs) (see details in Supplementary Table S1). Antibody-stained slides were exposed to the appropriate HRP-labeled polymer for 30 min and DAB chromogen (Agilent Dako, Santa Clara, CA, USA, Cat# GV82511-2) for 5 min. Slides were counterstained with hematoxylin (Fisher Scientific, Harris Modified Hematoxylin; Cat# SH30-4D), blued in 1% ammonium hydroxide (diluted from 30% ammonium hydroxide, Fisher Scientific, Pittsburgh, PA, USA, Cat# A669-500), dehydrated, and mounted with Permount (Fisher Chemical, Fairlawn, NJ, USA, Cat# SP15-100). Consecutive sections with omitted primary antibodies served as negative controls. The wash buffer used was (VWR Life Science, Solon, OH, USA, Cat# 0777-1L) 1xTBS with 0.05% tween20 (VWR Life Science, Solon, OH, USA, Cat# 0777-1L) Masson’s trichrome images were analyzed for densitometry using the Image-J software (National Institutes of Health, Bethesda, MD, USA, http://rsb.info.nih.gov/ij/; version 6.0). Slides stained for CD8-positive immunoreactivity and arginase-positive T-cells were scanned using an Aperio GT450 machine (Leica Biosystems; Vista, CA, USA) and images were analyzed using Aperio Image Scope software (version 12.4.3.5008).

### 2.7. Evaluation of Spleen Lymphocytes for Exhaustion Markers

Flow cytometry was performed using a FACSARIA IIu brand cell sorter (BD Biosciences, Franklin Lakes, NJ, USA) with 375 nm, 405 nm, 488 nm and 633 nm laser lines to measure the surface antibodies listed in Supplementary Table S2 [including CD4, CD3, CD8, CD279 (PD-1), CD274 (PD-L1), CD27, CD366 (TIM3), CD223 (LAG3), and CD272 (BTLA)]. Viability was determined using a pre-diluted Zombie Red^TM^ fixable viability solution (100 μL) (BioLegend, San Diego, CA, USA, Cat# 423109). One million viable lymphocytes were added to 5 mL Falcon clear tubes (Fisher Scientific, Emeryville, CA, USA, Cat# 352054), washed and re-pelleted, and the antibody master mix (50 μL) was added to suspend the cell pellets and incubated for 30 min for flow cytometry.

### 2.8. Measurement of T-Cell Activity by Cytokine Stimulation Assay

One million washed and viable lymphocytes were added to each well of a 6-well plate, with duplicate wells for each sample; one well serving as the control. The other wells were treated with 1 mL of cell activation cocktail [phorbol 12-myristate 13-acetate (PMA; 25 ng/mL) and ionomycin (1 µg/mL)] without Brefeldin A (BioLegend, San Diego, CA 500×; Cat # 423302). The 6-well plates were placed in the cell culture incubator at 37 °C overnight. The next day, 1 μL of Brefeldin A solution (BioLegend, 1000×, Cat# 420601) was added at 1 μL/1 mL to each treatment well to block Golgi release of cytokines so that they could be measured intracellularly by flow cytometry. The 6-well plates were placed in a cell culture incubator at 37 °C for 4 additional hours. The cells were then harvested and washed, followed by the addition of pre-diluted Zombie NIR™ (50 μL; BioLegend, San Diego, CA, USA) to the cells and incubated at room temperature in the dark for 20 min. The lymphocytes were then washed and stained with two surface antibodies [CD4 (Alexa Fluor 488; BioLegend, San Diego, CA. Cat # 100529) and CD8 (BV421; BioLegend, San Diego, CA. Cat # 100737)]. After 30 min of incubation, lymphocytes were washed with Cyto-Fast™ Perm Wash Solution and fixed with Cyto-Fast™ Fix/Perm Solution (BioLegend, Cat# 426803) at room temperature for 20 min. The lymphocytes were washed and stained with the intracellular cytokine antibodies listed in Supplementary Table S3 (including granzyme B, perforin, tumor necrosis factor-α, and interferon-γ) and incubated at room temperature in the dark for 20 min. The cells were washed again, resuspended in cell staining buffer (BioLegend, Cat# 420201) and subjected to flow cytometry. Flow cytometry data were acquired using FCS Express-6 software (version 6; De Novo Software, Glendale, CA, USA) and analyzed using FlowJo flow cytometry analysis platform (version 11; FlowJo LLC, Ashland, OR, USA).

### 2.9. Microbiome Analysis

High-throughput sequencing was performed by Transnetyx Inc. (Cordova, TN, USA) on fresh stool pellets collected from individual mice in control, proglumide, PD-1Ab, and combination therapy groups. Samples were collected before inoculation of cancer cells at 7 weeks of age and at the end of treatment at 12 weeks of age. Whole-genome 16s sequencing (WGS) was performed using an Illumina NextSeq 2000 instrument with fecal DNA extracted with a DNeasy extraction kit (Qiagen, Germantown, MD, USA, Cat# 47021). The results were analyzed by using a reference database [25] that includes 71,262 bacterial, 72,720 viral, 1821 fungal, 2231 archaeal, and 205 protozoan genomes (plus host genomes).

### 2.10. Statistical Analysis

Data were analyzed by both parametric and nonparametric methods. Student’s test, chi-square test, and Wilcoxon rank-sum test were used for group comparisons. The Shapiro–Wilk test was used to compare group differences for T-cell flow cytometry data. The histology changes seen in the TME were analyzed by one-way ANOVA and t-test with a Bonferroni correction for multiple comparisons to controls. Microbiome data analysis was assisted by the One Codex platform (Wilmington, DE, USA). For changes in individual bacterial genera, we used the formula (post-pre)/pre to calculate the change in the microbiome value from baseline. Three sources of microbiome data were analyzed: (1) number of reads from raw data; (2) estimated abundance from raw data; and (3) normalized data. Changes in each bacterium were analyzed using one-way ANOVA between all four treatment groups and Fisher’s LSD test to determine which specific means were significantly different from each other when the overall ANOVA result was significant. Graphics and statistics were analyzed using GraphPad Prism version 10.5.

## 3. Results

### 3.1. Effects of Proglumide and PD-1Ab Alone or in Combination on HCC Tumor Growth

**Figure 2:**
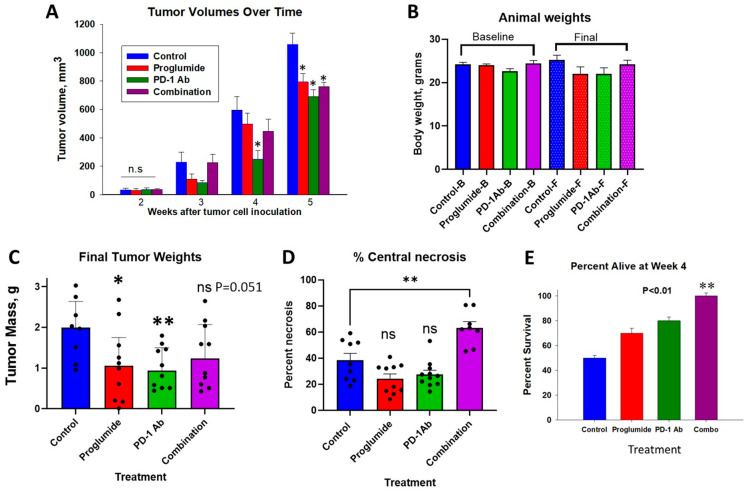
Tumor volumes over time, final tumor weights, and survival. (**A**) Tumor volumes are plotted for each weekly assessment over time and a final measurement was taken the day 34 at euthanasia prior to removal of tumors by dissection. Tumor volumes were equal at baseline in all treatment groups. Only the PD-1Ab-treated mice has significantly smaller tumors at week 3 compared to controls. At week 4 all treatment groups had smaller tumor volumes compared to volumes of control mice. Statistically significant compared to controls, * *p* < 0.05 Student’s t-test with Bonferroni correction. (**B**) Animal body weights in grams in each group at baseline and at the end of the study. (**C**) Final tumor weights at necropsy are shown for each treatment group compared to control tumors. Columns represent mean ± SEM for each group. Significant levels compared to controls include: * *p* < 0.05; and ** *p* < 0.01; Student’s t-test with Bonferroni correction. (**D**) Percentage of necrosis in tumor sections from each group shows a significant change from controls in the tumors of the combination-treated mice (** *p* < 0.01) and no significant difference in percentage of tumor necrosis was observed between control tumors and tumors from monotherapy-treated groups. (**E**) The percentage of mice alive after 4 weeks of treatment in 2nd cohort revealed that the combination-treated mice survived 50% longer than control mice bearing RIL-175 HCC tumors. Statistically significant compared to controls and to monotherapy ** *p* < 0.01 Student’s t-test with Bonferroni correction. (Image C reproduced and order modified with permission from *Int. J. Mol. Sci.* **2023**, *24*, 3625. https://doi.org/10.3390/ijms24043625) [26].

Tumor volumes were equal in all treatment groups at baseline, which was 14 days after tumor cell inoculation. Over the treatment period, mice treated with either proglumide or PD-1Ab monotherapy or with proglumide in combination with PD-1Ab had smaller tumor volumes than those in the control group (Figure 2A). There were no significant changes in animal body weights over the course of the study (Figure 2B).

All control and experimental animals were euthanized 35 days after tumor inoculation, because tumors in the control-treated mice reached the maximum size allowed by the IACUC protocol. One animal in the combination group died unexpectedly 3 days before euthanasia; this animal’s tumor was excised, weighed, and included in the results below, but its splenic cells were not used for surface receptors or cytokine re-stimulation. At necropsy, tumors were excised and weighed. The differences in the final tumor masses between the treatment groups are shown in Figure 2C. Compared to tumor mass of control mice, tumors of the treated mice decreased by 45% for proglumide; by 49% for PD-1Ab; and 35% for the combination treated mice (Figure 2C). Although smaller than controls, the mean weights of the combination-treated mice did not reach statistical significance (*p* = 0.051). Gross examination of the tumors from the combination-treated mice revealed fluctuance of the tumors and when sectioned, it was observed that these tumors had a large area of central necrosis that accounted for the swollen size and slight increase in weight compared to the tumors of the monotherapy-treated mice. The percentage of necrosis for each tumor section was analyzed on scanned images and compared between groups. Only the tumors of the combination treatment had significantly greater areas of central tumor necrosis compared to controls and compared to each of the monotherapy groups (Figure 2D). The appearance of an increase in tumor size or pseudo-progression is a known phenomenon that occurs with immune cell infiltration in response to immune checkpoint inhibitor therapy. The same treatments with mice bearing RIL-175 HCC tumors in another cohort showed that the mice treated with the combination therapy had a significant increased survival compared to controls and monotherapy mice (Figure 2E) suggesting the observed weight of the tumors was due to pseudo-progression from the immune response. ns = not significant.

### 3.2. Proglumide Therapy Decreases Tumoral Fibrosis and Alters the Immune Cell Signature

**Figure 3:**
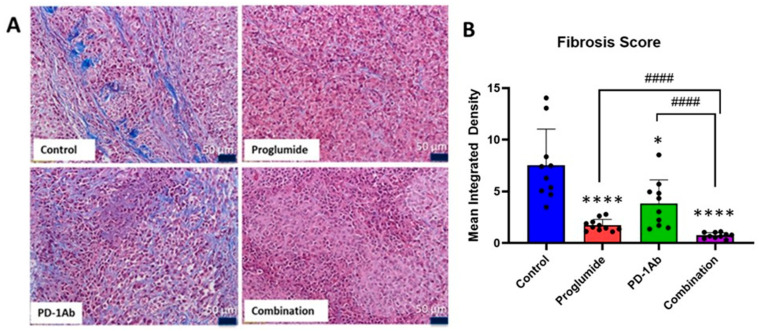
Proglumide and PD-1Ab therapy are synergistic in decreasing fibrosis in the tumor microenvironment. (**A**) Representative histological sections of tumors (Scale bar 50 µm) reacted with Masson’s trichrome staining of HCC tumors from control, proglumide, PD-1Ab and proglumide in combination with PD-1Ab treated mice are shown. (**B**) Computer analysis quantification demonstrated significantly decreased fibrosis in tumors of all treated mice, but a more pronounced effect was recorded in tumors of mice treated with proglumide or proglumide in combination with the PD-1Ab. Significantly different compared to control tumors * *p* < 0.05 and **** *p* < 0.0001 and significantly different than monotherapy compared to the combination #### *p* < 0.0001.

Masson’s trichrome staining showed extensive fibrosis of the HCC tumor microenvironment in control tumors. Less fibrosis was observed by Masson’s trichrome staining in the tumors from mice in all the treatment groups with the greatest difference observed in proglumide-treated mice (Figure 3A). Computer analysis by densitometry confirmed a statistically significant decrease in collagen staining compared to controls for each treatment group (Figure 3B) and a synergistic effect was noted in the tumors of the mice treated with both proglumide and the PD-1Ab (i.e., the combination) therapy group in comparison to the control and respective monotherapies (Figure 3B).

### 3.3. Proglumide Alters the Immune Cell Signature of the HCC Tumor Microenvironment

**Figure 4:**
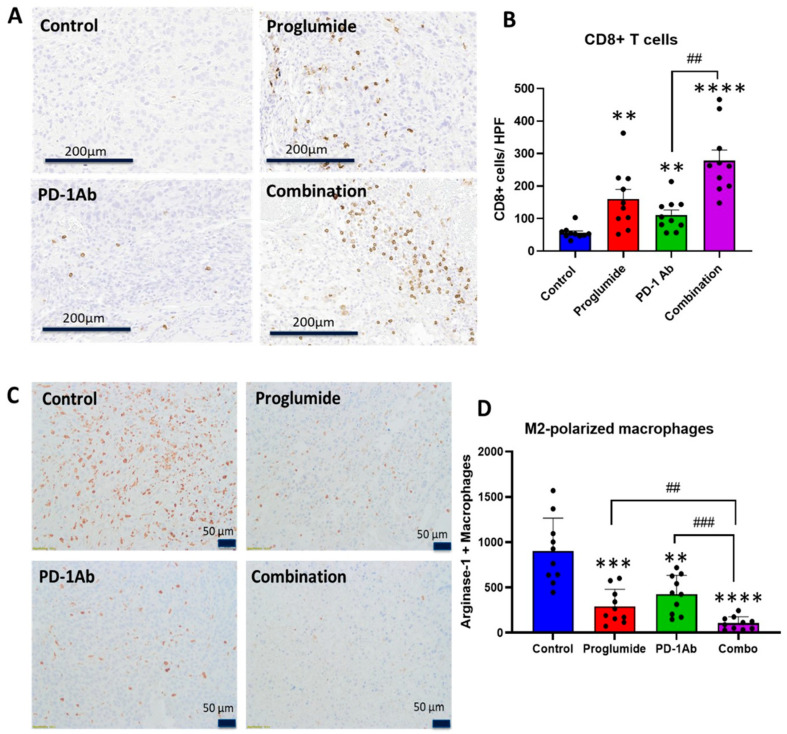
Proglumide in Combination with PD-1Ab therapy alters tumor immune cell signature. (**A**) Immunohistochemistry for CD8+ T-lymphocytes is shown from tumors representing each treatment group (scale 200 µm). (**B**) Analysis of cell counts demonstrates that CD8+ intratumoral lymphocytes are rare in tumors of control mice. CD8+ tumor infiltrating lymphocytes are significantly increased in mice treated with proglumide and PD-1Ab monotherapy. CD8+ cells are the greatest in mice treated with the combination of proglumide and PD-1Ab. (**C**) Representative images of arginase 1+ immunoreactive tumor-associated macrophages (TAMs) from tumors of each treatment group are shown (scale 50 µm). (**D**) The mean values ± standard error of the means was calculated from a computerized analysis of each histologic slide area from TAMs are shown. Significantly different from control ** *p* < 0.01; *** *p* < 0.005; and **** *p* < 0.0001. Significantly different between monotherapy and combination therapy ## *p* < 0.01 and ### *p* < 0.005.

The number of CD8+ T-cells per high-power field (HPF) was sparse in RIL control tumors. The mean number of CD8+ T-cells significantly increased in the tumors of mice treated with PD-1 Ab and proglumide; however, a greater number of CD8+ T-cells was observed in the tumors of mice treated with both drugs. Representative images from each treatment group are shown in Figure 4A. The mean number of CD8+ T-cells from each of the tumors was averaged per group. Compared to the number of CD8+ T-cells in control tumors, these immune cells increased in the tumors of proglumide-treated mice by 186%, 98% in the PD-1Ab-treated mice, and 396% in the tumors of mice treated with the combination therapy (Figure 4B). The number of CD8+ cells in the tumors of mice treated with the combination therapy was greater than the PD-1Ab monotherapy group (*p* < 0.01).

Immunosuppressive M2-polarized tumor-associated macrophages (TAMs) were abundant in the tumors of control mice. Representative images from each treatment group are shown in Figure 4C. The number of immunoreactive arginase+ M2-polarized TAMs was counted per HPF and plotted in Figure 4D. There were 3.1-fold fewer M2-polarized TAMs in the tumors of mice treated with proglumide and a 2.1-fold decrease in M2-polarized TAMs in tumors of mice treated with PD-1Ab monotherapy. However, the number of M2-polarized TAMs was significantly decreased by 8.7-fold in the tumors of mice treated with the combination regimen compared to controls suggesting a synergistic effect.

### 3.4. Combination Therapy Decreases T-Cell Exhaustion Markers

**Figure 5:**
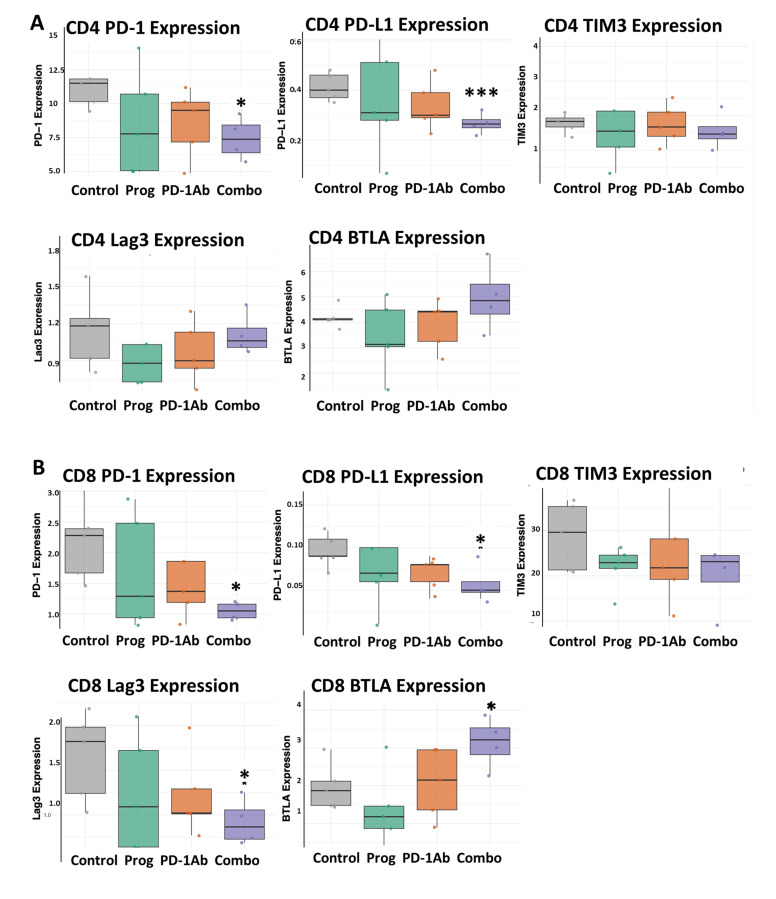
Expression of CD4+ and CD8+ T-cell exhaustion and surface markers by treatment group. (**A**) Shows the effects of PD-1Ab and proglumide monotherapy or combination on CD4+ T-cell surface receptors compared to the controls. (**B**) Shows the effect of the various treatments on the surface receptors of CD8+ T-cells. Additionally, a significant increase was observed in BTLA1 (*p* = 0.0366). Box plots show the expression levels of CD4+ and CD8+ T-cell exhaustion and surface markers from spleen lymphocytes of mice in each group. Boxes indicate the interquartile range (IQR), the horizontal line within each box denotes the median and the whiskers represent the range. Significantly different from controls * *p* < 0.05, and *** *p* < 0.005.

Tumors evade the effects of immune checkpoint antibodies through a variety of mechanisms. High levels of PD-1 (or CD279) expression on tumor-infiltrating lymphocytes (TILs) are often associated with T-cell exhaustion [33]. The presence or absence of PD-L1 (or CD274) expression on tumors is known to be an important predictor of response to ICIs and is typically upregulated on T-cells in cancer in response to antigen presentation [34]. Tumors positive for PD-L1 generally show higher responses to ICIs; however, regulating the activity of both CD4+ and CD8+ T cells, influencing T-cell dysfunction and T-cell infiltration. We examined the expression of PD-1 and PD-L1 surface receptors on both CD4 and CD8 T-cells isolated from mouse spleens treated with the above therapies compared to controls. T-lymphocytes were isolated from spleen mononuclear cells of mice that had been treated with proglumide, PD-1Ab, and the combination of proglumide and PD-1Ab compared to controls, as shown in Figure 5. PD-1 and PD-L1 expression on CD4 (Figure 5A) and CD8 T-cells (Figure 5B) decreases significantly in spleen cells isolated from mice treated with the combination of proglumide and PD-1Ab. Monotherapy with PD-1 or proglumide alone did not significantly alter the expression of the surface markers of CD4 or CD8 T-cells. However, the combination treatment of PD-1Ab and proglumide resulted in a significant reduction in PD-1 (*p* = 0.012) and PD-L1 (*p* = 0.003) in CD4 T-cells (Figure 5A). Combination therapy also significantly decreased PD-1 (*p* = 0.016), PD-L1 (*p* = 0.034) and Lag3 (*p* = 0.028) expression in CD8+ T-cells (Figure 5B). Although BTLA expression is more commonly a marker for B-cells, we examined its expression and found it to be increased in CD8 T-cells in mice treated with the combination therapy. While CD4 and CD8 T-cell exhaustion share some features, CD4 T-cell exhaustion is less understood, particularly in terms of its progression, subset-specific impacts, and therapeutic potential.

### 3.5. Improved Cytokine Release from T-Cells Treated with Proglumide

**Figure 6:**
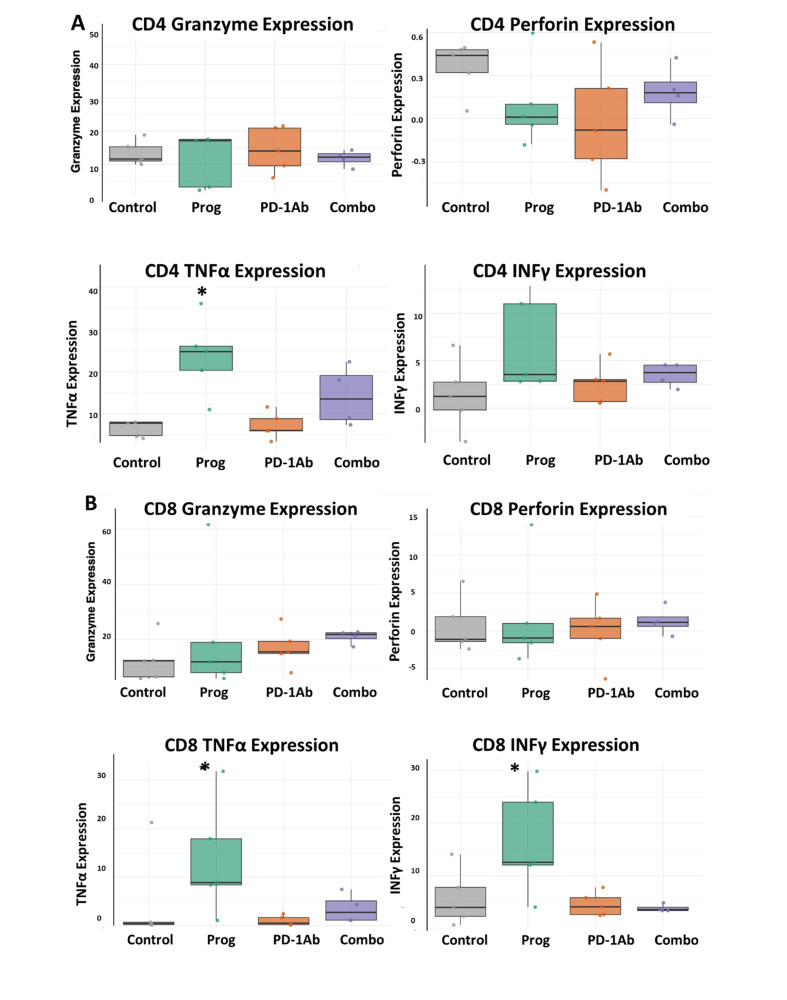
Cytokine release from T-cells from treatment groups compared to controls. (**A**) Cytokine release from mouse CD4 T-cells is shown for each treatment group compared to controls. Cytokines measured included granzyme, perforin, TNFα, and INFγ. (**B**) Cytokine release from CD8 T-cells after stimulation for granzyme, perforin, TNFα, and INFγ is shown. Box plots show the expression levels of CD4+ and CD8+ T-cell cytokine release from spleen lymphocytes of mice in each group. Boxes indicate the interquartile range (IQR), the horizontal line within each box denotes the median and the whiskers represent the range. * *p* < 0.05.

CD8 T-cell exhaustion leads to reduced cytotoxic activity, loss of proliferative capacity, and altered cytokine production. CD4 T-cell exhaustion affects helper functions, cytokine production, B-cell support, and macrophage activation. T-cell exhaustion is characterized by a reduction in perforin, granzyme-B, IFNγ, and TNFα levels, resulting less responsiveness to immune checkpoint therapy [35]. A cytokine release assay was performed to observe the effects of treatments on the function and activity of T-cells after stimulation. Isolated splenic T-lymphocytes were obtained from mice treated with proglumide, PD-1Ab, and combination therapy of proglumide and PD-1Ab to determine whether treatment enhances the ability of T-cells to release cytokines. Figure 6A shows the results of the cytokine release assay in CD4+ T-cells. Only CD4 T-cells from proglumide-treated mice exhibited increased TNFα cytokine levels after stimulation. There were no significant differences in granzyme, perforin, and IFNγ cytokine release from CD4 T-cells between the control, PD-1Ab, and combination therapy-treated mice. Cytokine release from CD8 T-cells is shown in Figure 6B. Proglumide treatment significantly increased the levels of TNFα (*p* = 0.032) from CD8+ T-cells compared with PD-1Ab. Proglumide-treated mice also exhibited an increased release of IFNγ (*p* = 0.032) in CD8+ T-cells compared to the combination-treated mice.

### 3.6. Combination Therapy with Proglumide and PD-1Ab Alters the Mouse Microbiome

Alpha diversity metrics summarize the structure of a microbial community in terms of its richness (number of taxonomic groups), evenness (distribution of abundances of the groups), or both [36]. We examined the alpha diversity of our microbiome data using Shannon’s index and found stable data for the pretreatment samples (Supplementary Figure S2A) and post-treatment samples (Supplementary Figure S2B). A Principal Coordinate Analysis (PCA) plot was generated from the beta diversity distance matrix to visualize the differences in microbial community composition (beta diversity) between samples for pretreatment (Supplementary Figure S3A) and post-treatment samples (Supplementary Figure S3B). The percentage of variation in the beta diversity of the microbiome explained by the first principal coordinate (PC1) was 78.53% in the pretreatment samples and 44.84% in the post-treatment samples, implying that there was more variation in the samples before therapy. Principal component 2 (PC2) of beta diversity measures the dissimilarity in microbiome composition between samples. PC2 increased from 7.88% in the pretreatment samples to 25.55% in the post-treatment samples, suggesting that the treatment induced a significant shift in the patterns of variation within the microbial communities. The shift in the explained variance suggests that the treatment significantly affected the overall structure and composition of the microbiome.

**Figure 7:**
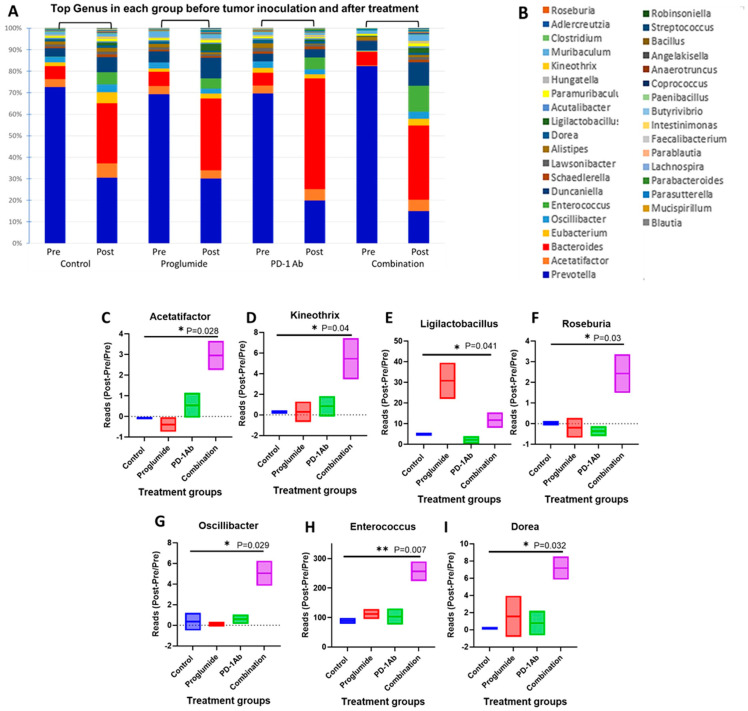
Combination therapy alters the gut microbiome. Microbiome analysis of mouse feces by whole-genome 16s sequencing of mice is shown in control, proglumide, PD-1Ab and proglumide in combination with the PD-1Ab before and after the treatment period. (**A**) The mean number of reads represented as percentage in stacked columns for the top 36 bacteria genus for each group is shown pre-treatment and post-treatment. (**B**) A list of the represented genus by name with color coding is shown and correlates with the figure in A. Significant changes in bacteria from baseline values compared to after treatments are shown for the following bacteria genus: (**C**) Acetatifactor; (**D**) Kineothrix; (**E**) Ligilactobacillus; (**F**) Roseburia; (**G**) Oscillibacter; (**H**) Enterococcus; and (**I**) Dorea. Significant differences between the groups by one-way ANOVA * *p* < 0.05, ** *p* < 0.01. The dotted line in C, D, F, and G shows the zero value.

Several beneficial bacterial taxa were significantly increased in the feces of mice treated with proglumide and combination therapy. The top 36 bacteria genera are shown at baseline (prior to tumor inoculation or treatment) and the same bacteria are shown after the treatments and before necropsy for each group (Figure 7A). A list of the genera represented by names with color coding is shown (Figure 7B). The microbiome composition was similar in the pretreatment fecal samples from all cohorts and the most abundant bacterial genus is shown in Supplementary Figure S4. Prior to treatment, the fecal microbiome across all groups was dominated by Gram-negative, anaerobic Prevotella, a genus associated with increased expression of microbiome-derived inflammatory products and more abundant in patients with advanced fibrosis [37]. By the end of the treatment, Prevotella levels had decreased across all groups. Previous studies have shown that depleting Prevotella improves survival in liver cancer [38]. Although the control mice did not receive treatment, there was a change in the microbiome compared to the baseline. These changes can be attributed to the change in diet, the change in environment, aging of the mice, or the fact that the mice did not have HCC at when the initial microbiome sample was collected. Significant changes in the microbiota were identified when comparing the pre-inoculation samples to the post-treatment samples when normalized to the controls. Several bacteria that are beneficial in improving the response to immunotherapy were significantly increased when proglumide and PD-1Ab were used in combination therapy (Figure 7C–I). The genera Acetatifactor, Kineothrix, and Oscillibacter, classified as gram-variable, Gram-positive, and Gram-negative, respectively, were also increased in the microbiome of combination-treated mice compared to controls. Although relatively new to microbiome research, their presence may indicate additional immunomodulation to further enhance ICIs therapy [39]. Dorea, another Firmicutes genus that increased in combination-treated mice, has been associated with fewer exhausted T-cells and an improved response to immune checkpoint inhibitors [40]. Studies have shown that Enterococcus can release stimulatory molecules, such as muramyl dipeptide (MDP) fragments, which activate the innate immune sensor NOD2. This activation enhances immunotherapy responses by directly activating macrophages, reprogramming monocytes, generating dendritic cells, and priming them for cross-presentation to CD8+ T-cells [39]. Roseburia, a bacterium that has been shown to improve anti-PD-1 efficacy by inducing functional CD8^+^ T-cells [41], was significantly increased with the combination therapy. Lactobacillus species, known to promote T-cell and immune cell activation through the release of pro-inflammatory cytokines and short-chain fatty acids (SCFAs), can enhance immune checkpoint inhibitor therapy outcomes. SCFAs, produced by beneficial gut microbiota, are linked to CD8+ T cell activation and function, increased circulating cytotoxic T lymphocytes, and reduced Treg proliferation [42]. Ligilactobacillus, a SCFA-producing Lactobacillus, is known for its immunomodulatory effects, including TNFα induction which is consistent with the observed increase in TNFα following proglumide treatment [43,44].

## 4. Discussions

In this investigation, we found that combination therapy with proglumide and a PD-1Ab altered the HCC tumor microenvironment by decreasing fibrosis and M2-TAMs, and increasing the influx of CD8+ T-cells into the tumors, rendering the tumors more responsive to immune checkpoint antibody therapy. The combined treatment exhibited a greater than additive effect on remodeling the TME and immune cell signature, or synergistic effect. Combination therapy also resulted in a decrease in T-cell exhaustion markers and a greater response to release pro-inflammatory cytokines in both activated CD4+ and CD8+ T-cells. Another unique finding from the present investigation was that the combination of proglumide and PD-1Ab also altered the gut microbiome in mice, increasing the number of beneficial bacteria to boost the efficacy of cancer immunotherapy in HCC patients.

Although diminished tumor volumes were observed in all treatment groups, surprisingly, we did not find a decrease in the final tumor weights at necropsy in the combination-treated mice. However, when the tumors were paraffin-embedded and stained with H&E, it was noted that the larger size was due to central tumor necrosis. Pseudo-progression is a phenomenon that causes an initial increase in tumor size in response to therapeutics before tumor shrinkage. It is a well-studied occurrence with immune checkpoint antibody therapy owing to activated T-cell recruitment to the tumor and increased inflammation. Pseudo-progression is often observed in tumors with necrosis and edema [45,46,47]. Consistent with literature, since the mice treated with the combination therapy exhibited increased CD4+/CD8+ pro-inflammatory cytokines and an abundance of infiltrating CD8+ T-cells within the tumor microenvironment, this change resulted in necrosis and pseudo-progression. Increased tumor size from the inflammatory response typically occurs before tumor shrinkage. All of the mice in this experiment were necropsied on the same day because the size of the control tumors had reached the allowed capacity by IACUC and we wanted the same time point for analysis of T-cells and microbiome. As demonstrated in another cohort treated with the same regimen this pseudo-progression is associated with improved survival as previously described [26].

It has been established that a “cold” tumor microenvironment composed of dense fibrosis and minimal CD8+ T-cells predicts a poor response to immune checkpoint therapy [48,49]. An exciting finding in our study was that proglumide alone or in combination with PD-1Ab therapy resulted in a significant reduction in tumoral fibrosis. Hepatic fibrosis modulates the activity of inflammatory cells in the liver by reducing the infiltration of T-cells, contributing to tumor progression [50]. We believe that the decrease in fibrosis and remodeling of the tumor microenvironment are responsible for the influx of CD8+ T-cells. More CD8+ lymphocytes within tumors have been associated with better treatment responses and overall survival in patients with HCC [51].

M2-polarized macrophages render tumors less responsive to therapy, and in this investigation, we observed that the number of M2-polarized TAMs significantly decreased in the treated mice compared to controls. *ELANE* is a gene that codes for neutrophil elastase and influences the polarization of macrophages in the TME. We previously demonstrated that proglumide treatment prevents the release of cancer-derived neutrophil elastase-rich exosomes in the TME (abstract, APS April 2025), thus providing an explanation for the changes noted in this investigation with fewer M2-polarized TAMs in tumors of proglumide-treated mice.

T-cell exhaustion is a dysfunctional state characterized by a hierarchical loss of effector functions and proliferation [52]. Our investigation demonstrated that another mechanism implicated in the beneficial response to combined therapy included a reduction in T-cell exhaustion markers and improved cytokine release. The pro-inflammatory cytokines TNFα and IFNγ can improve tumor responsiveness to immune checkpoint inhibitors by enhancing T-cell infiltration and activation within the tumor [53,54]. Notably, proglumide treatment enhanced specific cytokine responses, specifically increasing TNFα in CD4+/CD8+ T-cells compared to PD-1Ab monotherapy. These findings suggest that proglumide may prevent T-cell exhaustion and reinvigorate surrounding immune cells, rendering them more responsive to immune checkpoint inhibition.

Recent studies have shown that several bacterial genera within the gut microbiome can enhance antitumor responses to immune checkpoint inhibitors in mice. Moreover, an increase in beneficial bacteria can improve the efficacy of anti-PD-1 therapy by stimulating immune cell activity in mouse tumors. Bile acids play an important role in determining the abundance, diversity and metabolism of the gut microbiome [55]. Proglumide and another CCK receptor antagonist, loxiglumide have been shown to stimulate bile acid flow [56]. Intestinal motility can also alter the microbiome. CCK peptide stimulates motility of the gastrointestinal tract [57], and CCK receptor antagonists have been shown to slow intestinal motility [58]. Proglumide was originally developed years ago for the treatment of gastric ulcers [59] and a reduction in gastric acid may also contribute to changes in the intestinal bacterial flora.

ICIs are associated with off-target toxicity [60] including colitis, dermatitis, thyroiditis, nephritis, hepatitis, pneumonitis and adrenal insufficiency [61]. Counter therapies with agents such as infliximab that block inflammatory cytokines, i.e., TNF-α, are being tested to prevent ICI-induced colitis [62]. Elevated liver-associated enzymes in subjects receiving ICIs or chemotherapy could represent reactivation of hepatitis B in a hepatitis B core Ab+ subject or may also be due to ICI-induced hepatitis [63]. Although not as common as the ICI-induced inflammation in other organs, pancreatitis has also been reported as a potential side effect of ICI therapy [64]. Proglumide has a broad safety profile, and has been shown to decrease tissue inflammation, lower transaminases in hepatitis [65], and decrease pancreatitis as demonstrated by lowering serum lipase and C-reactive protein levels [66]. Furthermore, proglumide exhibits a direct anti-tumor effect by blocking upstream PI3K-AKT-mTOR signaling pathways through antagonism of the CCK-BR perhaps allowing for the use of a lower dose of ICI with fewer immune mediated side effects. Proglumide has demonstrated efficacy and safety and several recent clinical trials (Table 1); therefore, the addition of proglumide to immune-mediated therapies may not only improve efficacy but also decrease the toxicity observed with ICIs.

One possible shortcoming of our work is that we only used one dose of PD-1Ab, (50 µg). It is possible that if a higher dose was used, a more pronounced effect with the PD-1Ab monotherapy would have been observed. However, several doses of PD-1Ab were pre-tested, and this dose exhibited the best synergy with proglumide in HCC and showed a significant survival benefit in combination-treated mice over monotherapy [26]. In the clinic, it would be beneficial if the efficacy of ICI could be improved without escalating the dose, because higher doses are associated with more off-target toxicity.

## 5. Conclusions

New strategies are needed to improve therapy for advanced hepatocellular carcinoma. Various approaches have been used to improve the efficacy of immune checkpoint antibodies [68]. The novelty of proglumide is that it improves the response to ICIs by more than one mechanism and has been demonstrated to be safe in human subjects with liver disease and cancer. Combining proglumide with an immune checkpoint inhibitor may improve the response and survival of patients with HCC.

## 6. Patents

Georgetown University owns a licensed patent (US17/678,754) titled, “Treating cancer with a CCK receptor inhibitor and an immune checkpoint inhibitor” and Dr Smith is a co-inventor.

## Figures and Tables

**Table 1:** Clinical trials with proglumide.

Clinical Trial	Registration Number	Outcome	Publication
Metabolic dysfunction-associated steatohepatitis, MASH	NCT04152473	Decreased transaminases, less fibrosis by FibroScan	[65]
Cirrhosis pharmacokinetic and safety study	NCT04814602	Renal excretion, no changes in liver function	[67]
Chronic pancreatitis	NCT05551858	Decreased pain, C-RP, lipase	[66]
Pancreatic cancer	NCT05827055	Safe with chemotherapy, decreased fibrosis and altered immune cell signature in tumor	(AACR abstract, Boston September 2025)

## Data Availability

The data generated in this study are available in the article and its supplementary files. Microbiome sequencing data are available at: https://doi.org/10.5281/zenodo.16574017.

## Supplementary Materials

The following supporting information can be downloaded at: https://www.mdpi.com/article/10.3390/cancers17182998/s1, Table S1: Antibodies used for tumor immunohistochemistry; Table S2: Antibodies employed for T-cell Surface Receptor Staining for Flow Cytometry; Table S3: Antibodies Employed for Cytokine and Re-stimulation Analyses; Figure S1: Pilot dosing and cell inoculation experiments; Figure S2: (A) Alpha diversity of fecal samples pretreatment. (B) Alpha diversity of fecal samples post-treatment; Figure S3: (A) Beta diversity for the microbiome samples is shown pretreatment and (B) Beta diversity for the microbiome samples is show for the post-treatment samples; Figure S4: Graphical representation of the most abundant bacteria (genus) in all the mice at Baseline before tumor inoculum or treatment. Prevotella is shown on a separate graph since the magnitude of this bacteria in # of reads was much higher than the other bacteria at Baseline.

## Abbreviations

The following abbreviations are used in this manuscript:

CCK	cholecystokinin
CCK-BR	cholecystokinin-B receptor
HCC	hepatocellular carcinoma
HPF	high-power field
ICI	immune checkpoint inhibitors
IFNγ	interferon-gamma
PD-1 Ab	programmed death-1 antibody
SCFAs	short-chain fatty acids
TAMs	tumor-associated macrophages
TME	tumor microenvironment
TNFα	tumor necrosis factor-alpha
