# Supplementary material for: Mechanistic Insights into Proglumide’s Role in Immune Cell Efficacy and Response to Immune Checkpoint Inhibitor Therapy in Hepatocellular Carcinoma
Source: Cancers (Basel). 2025 Sep 14;17(18):2998. doi: 10.3390/cancers17182998 (PMC12468770; doi:10.3390/cancers17182998)
Supplement: Supplementary file 1 [file cancers-17-02998-s001.zip › cancers-3851912-supplementary.pdf]

SUPPLEMENTARY DATA

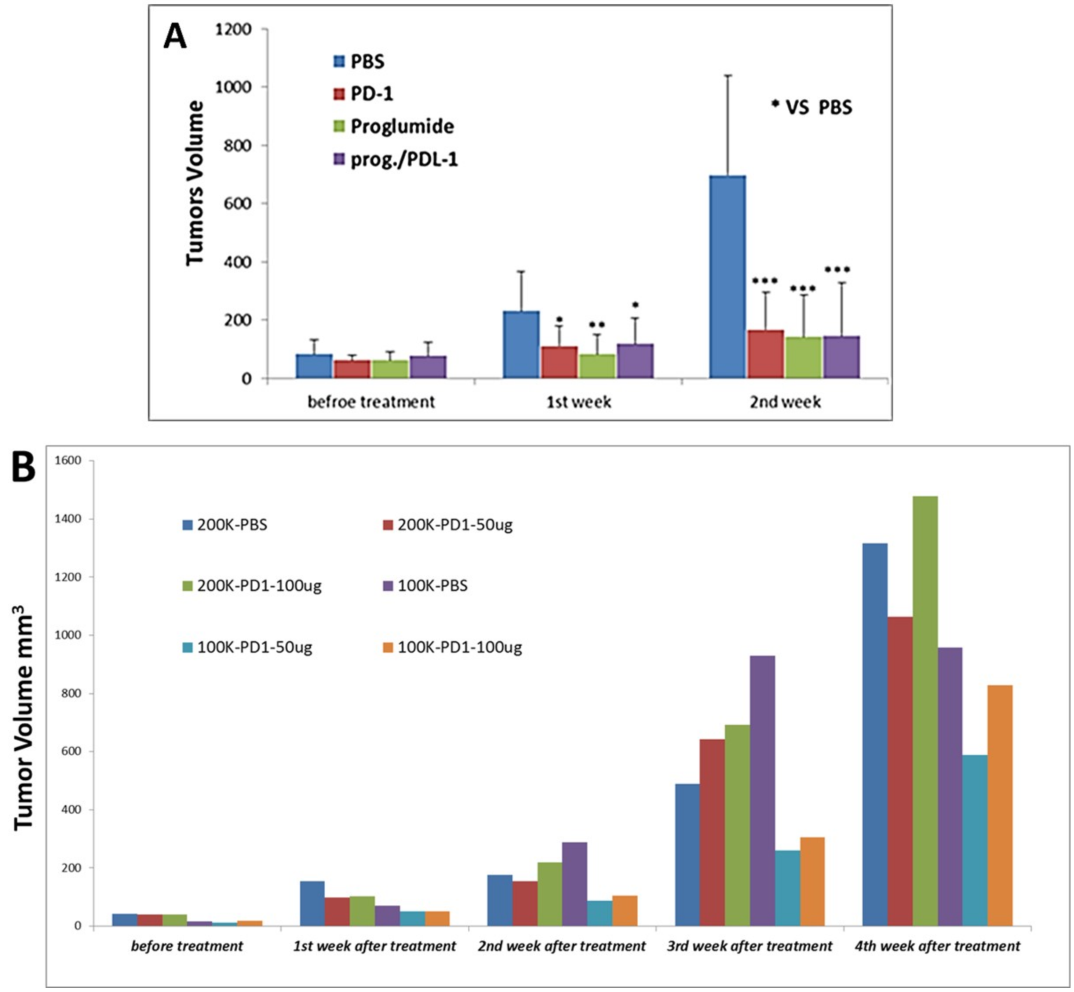

Supplementary Figure S1 Pilot dosing and cell inoculation experiments. (**A**) In the first pilot experiment, 700,000 RIL-175 cells were injected subcutaneously into N=20 C57BL/6 mice on both the right and left flank. Tumors were palpable in all mice within 7 days and mice were divided into 4 treatment groups (N=5 mice/group- or N=10 tumors per group). Treatment included the following: PBS (controls), PD-1 antibody (Ab) 150 µg x 3 (4 days apart), proglumide (0.1 mg/ml in the drinking water), or a combination of PD-1 Ab and proglumide. The tumors were measured with calipers at baseline and each week. After only 2 weeks the control tumors were very large and the experiment was terminated. (**B**) In the second series of experiments mice were inoculated with either 200,000 or 100,000 RIL-175 cells into the right and left flank subcutaneously to compare. Tumors of the 200,000 cells are depicted in the left columns and tumor volumes from the lower cell inoculum are depicted in the right columns. These tumors grew more slowly than the prior experiment with 700,000 cells injected. Mice treated with the 50µg PD-1Ab dose in both cohorts had smaller tumors than those mice treated with the moderate (100µg) PD-1Ab dose. Overall, these tumors grew more slowly and the 50µg PD-1Ab dose appeared to be the optimal dose to combine with proglumide for the current study.

Antigen	Supplier	Cat#	Titer	Source/ Isotype
CD8α (D4W2Z) XP®	Cell Signaling Technology	#98941	1:50	Monoclonal Rabbit IgG
Arginase 1	ThermoFisher Scientific	PA5-29645	1:1,800	Rabbit polyclonal

Supplementary Table S1: Antibodies used for tumor immunohistochemistry.

Fluorescent Label	Antigen	Supplier	Cat#	Clone
PE	CD3	Cytek Biosciences (Fremont, California, United States of America)	50-0032-U100	17A2
Alexa Fluor 488	CD4	BIOLEGEND® (San Diego, California, United States of America)	100529	RIM4-5
BV421	CD8	eBioscience™ (San Diego, California, United States of America)	404-0081-82	53-6.7
BV711	CD279 (PD-1)	BIOLEGEND®	135231	29F.1A12
PE- Fire 640	CDDorea274 (PD-L1)	BIOLEGEND®	124346	10F.9G2
APC-Fire 750	CD27	BIOLEGEND®	124238	LG.3A10
BV605	CD366 (TIM3)	BIOLEGEND®	119721	RMT3-23
APC	CD223 (LAG-3)	Cytek Biosciences	20-2231-U100	C9B7W
PE-Cy7	CD272 (BTLA)	BIOLEGEND®	139116	6A6

Supplementary Table S2: Antibodies employed for T-cell Surface Receptor Staining for Flow Cytometry

Fluorescent Label	Antigen	Supplier	Cat#	Clone
Pacific Blue	Granzyme B	BIOLEGEND®	515408	GB11
PE	Perforin	BIOLEGEND®	154306	S16009A
PE-eFluor 610	TNFα	eBioscience™	61-7321-82	MP6-XT22
APC	IFNγ	Cytek Biosciences	20-7311-U100	XMG1.2

Supplementary Table S3: Antibodies Employed for Cytokine and Re-stimulation Analyses

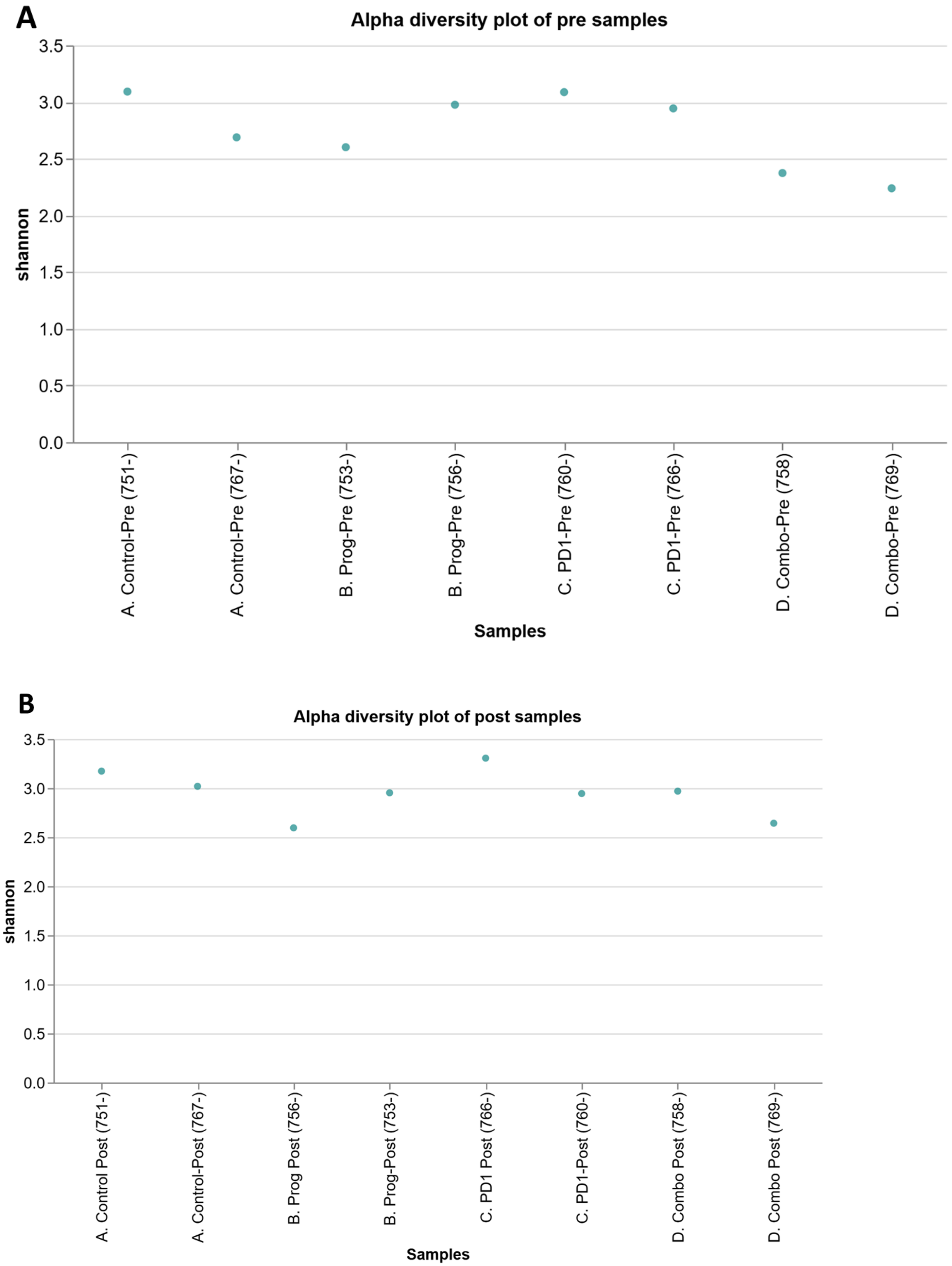

Supplementary Figure S2 (**A**) Alpha diversity of fecal samples pretreatment. (**B**) Alpha diversity of fecal samples post-treatment.

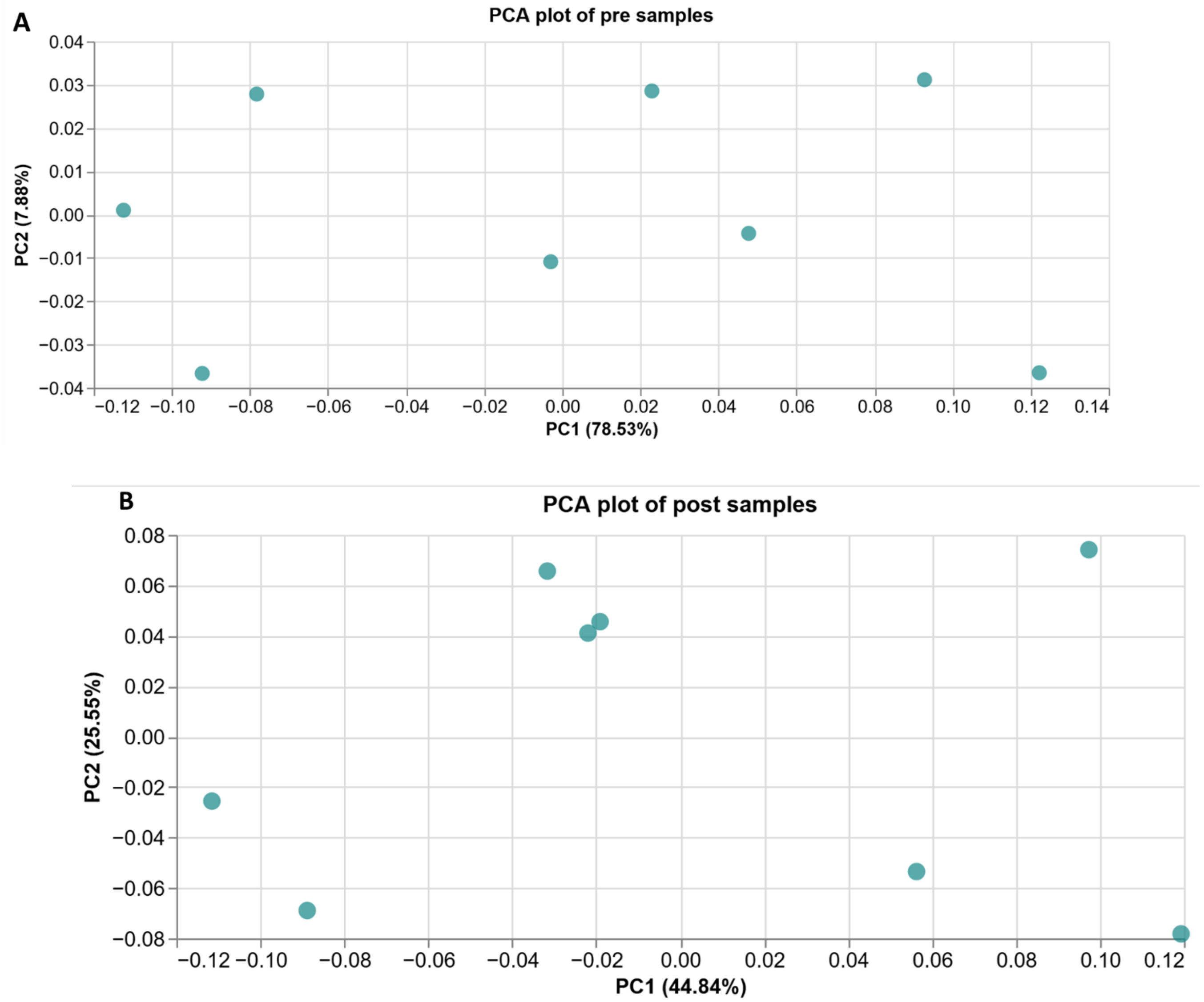

Supplementary Figure S3. (**A**) Beta diversity for the microbiome samples is shown pretreatment and (**B**) Beta diversity for the microbiome samples is show for the post-treatment samples.

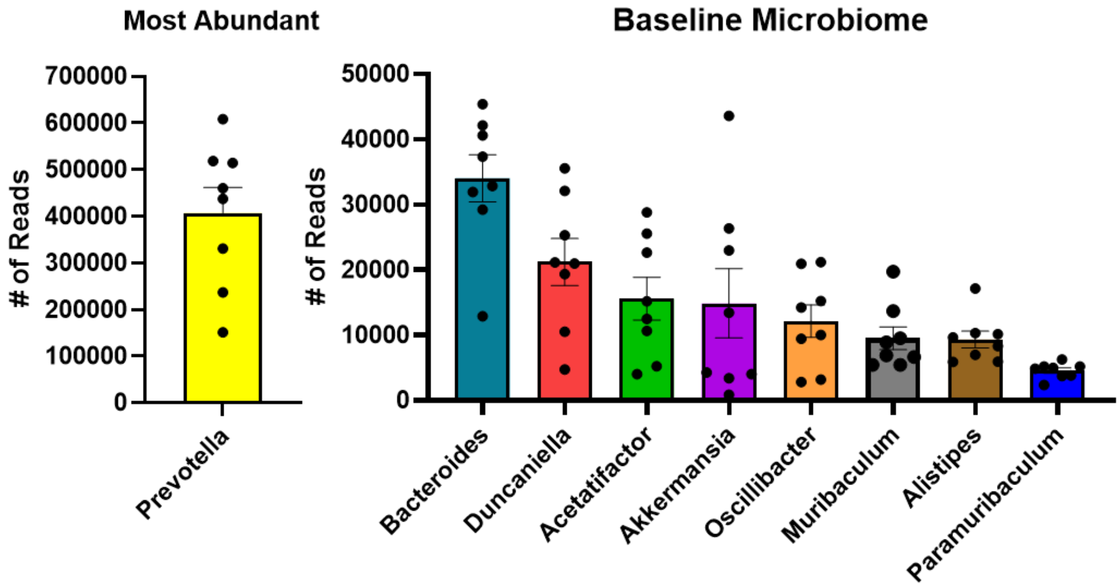

Supplementary Figure S4. Graphical representation of the most abundant bacteria (genus) in all the mice at Baseline before tumor inoculum or treatment. Prevotella is shown on a separate graph since the magnitude of this bacteria in # of reads was much higher than the other bacteria at Baseline.
